# Feasibility and Use of the Mobile Food Record for Capturing Eating Occasions among Children Ages 3–10 Years in Guam

**DOI:** 10.3390/nu7064403

**Published:** 2015-06-02

**Authors:** Tanisha F. Aflague, Carol J. Boushey, Rachael T. Leon Guerrero, Ziad Ahmad, Deborah A. Kerr, Edward J. Delp

**Affiliations:** 1 Human Nutrition, Food, and Animal Sciences University of Hawaii at Mānoa, Honolulu, HI96822, USA; E-Mail: franquez@hawaii.edu; 2 Epidemiology Program University of Hawaii Cancer Center, Honolulu, HI96813, USA; 3 College of Natural and Applied Sciences University of Guam, Mangilao, GU 96923, USA; E-Mail: rachaeltlg@uguam.uog.edu; 4 School of Electrical and Computer Engineering Purdue University, Lafayette, IN 47907, USA; E-Mails: zahmad@purdue.edu (Z.A.); ace@ecn.purdue.edu (E.J.D.); 5 School of Public Health, Curtin University, Bentley, Perth WA 6102, Australia; E-Mail: d.kerr@curtin.edu.au

**Keywords:** mobile food record, children, dietary assessment, Pacific Islanders

## Abstract

Children’s readiness to use technology supports the idea of children using mobile applications for dietary assessment. Our goal was to determine if children 3–10 years could successfully use the mobile food record (mFR) to capture a usable image pair or pairs. Children in Sample 1 were tasked to use the mFR to capture an image pair of one eating occasion while attending summer camp. For Sample 2, children were tasked to record all eating occasions for two consecutive days at two time periods that were two to four weeks apart. Trained analysts evaluated images. In Sample 1, 90% (57/63) captured one usable image pair. All children (63/63) returned the mFR undamaged. Sixty-two children reported: The mFR was easy to use (89%); willingness to use the mFR again (87%); and the fiducial marker easy to manage (94%). Children in Sample 2 used the mFR at least one day at Time 1 (59/63, 94%); Time 2 (49/63, 78%); and at both times (47/63, 75%). This latter group captured 6.21 ± 4.65 and 5.65 ± 3.26 mean (±SD) image pairs for Time 1 and Time 2, respectively. Results support the potential for children to independently record dietary intakes using the mFR.

## 1. Introduction

Traditional dietary assessment methods for children are challenging related to literacy levels, limited cognitive abilities, and difficulties estimating portion size at different developmental stages [1]. The day-to-day composition of young children’s diets varies reflecting changing and developing taste and flavor perceptions [2]. Measuring dietary intake in children provides insight into food choices and eating habits, like fruit and vegetable consumption, which is lowest in children compared to adults in the United States (US) [3,4]. A systematic review of dietary assessment methods in children found the most accurate method for reporting energy intake among 4 to 11 year olds was the multiple pass 24 h dietary recall as reported by the parent [5]. For children aged 0.5 to 4 years, the authors concluded that the weighed food record provided the best estimate for energy intake [5]. However, due to the burden associated with keeping weighed food records, they are less useful in community dwelling populations [6]. Additionally, many young children have multiple eating occasions in different settings outside of home, such as childcare centers, or schools where parents are not the primary informants in all settings [7]. Therefore, proxy dietary assessments reported by parents may not be as accurate as desired.

New self-reported methods shown to be useful for adolescents, 11–18 years old, are image-based dietary assessments using mobile devices, such as the mobile food record (mFR) [8,9]. The mFR is a dietary record application that uses the embedded camera in a mobile device (e.g., mobile telephone, iPod) to record dietary intake. Images can enhance self-reported data and, in this case, provide the primary record of dietary intake to obtain valid estimates of energy intake. Briefly, methods of automated image analysis or a trained analyst can be used to identify the food in the image and estimate volume of food consumed [8,10,11]. In addition to real-time data collection, this method eliminates reliance on the respondent’s memory, proxy reports, and ability to write and/or estimate portions [8]. Thus, the usability of the mFR with young children is worthwhile to examine.

The current generation of children, born into the digital age, lends to a high level of technology readiness. Use of technologies, such as web- and mobile-based applications may address many of the barriers to gathering accurate dietary data from children. Research involving children will contribute to research to advance mFR technology by addressing age-specific developments. This will allow the mFR to serve as a more accurate and feasible method of dietary assessment for children.

The overall goal of the analyses described in this paper was to determine if children 3 to 10 years could successfully use the mFR, previously tested with 11 to 18 year olds [8,9]. The first sample of children was evaluated using the mFR on three defined skill sets: (1) capturing a usable image pair; (2) demonstrating responsibility for the mobile device; and (3) reporting the feasibility and usability of using the mFR. A usable image pair is an image taken before an eating occasion that includes foods and/or beverages and the fiducial marker, as well as an image taken after the same eating occasion including an appropriate scene (e.g., an empty plate or food wrapper) and the fiducial marker. The second sample of children was assessed for cooperation using the mFR to capture usable image pairs of eating occasions over two days during two time periods as a method to collect fruit and vegetable intake among community-dwelling children (3 to 10 years).

## 2. Methods

Data were collected from two samples of boys and girls registered in preexisting summer day camps in Guam [10]. For the analyses reported here, only those children between 3 and 10 years old were included. These ages represent ages for which no previous studies have examined use of the mFR. All summer programs were open to children of all race/ethnic groups. Recruitment materials for the studies were made available in the registration areas or the camp registration packets. Children were recruited during drop-off or pick-up times. Informed consent was obtained from the parents and assent from their children. The Human Studies Program of the University of Hawaii and the University of Guam Committee on Human Subjects Research approved the study methods described here.

For the first sample (Sample 1) children were recruited from two summer day camps in 2013: A cultural immersion camp for 3 to 12 year olds and a recreational sports camp for children 5 to 15 years old [10]. Participants from these camps were tasked to use the mFR to take an image pair of, at least, one eating occasion on one day while at camp. Children were in possession of the mFR from the time after instruction and during, at least, one eating occasion, plus the time in between, and variable amounts of time after. Upon returning the mFR to researchers, participants were asked to complete a brief questionnaire on feasibility and usability of the mFR and fiducial marker (FM).

**Figure 1:**
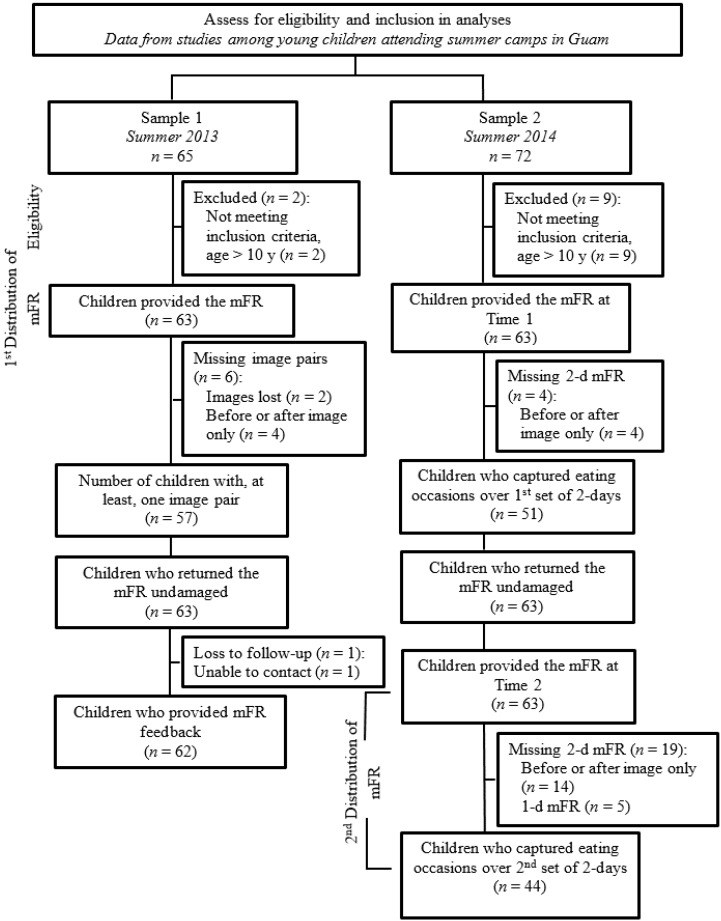
Assessment of eligibility for inclusion in analyses among children attending summer day-camps in Guam during summer 2013 (Sample 1) and during summer 2014 (Sample 2).

During 2014, the second sample of children was recruited from the same cultural immersion camp described in Sample 1 and a university-based day camp for children 6 to 12 years. Participants were tasked to capture image pairs of all eating occasions over two-days during two time periods for a total of 4 days. The data collected from the mFR was used to assess whole fruit and vegetable intake before (±2 weeks) and after (±1 week) the summer day camp (*i.e.*, Time 1 and Time 2). Data from both time periods were included in this analysis. Figure 1 shows the data collection flow for the two samples.

### 2.1. Description of the mFR

**Figure 2:**
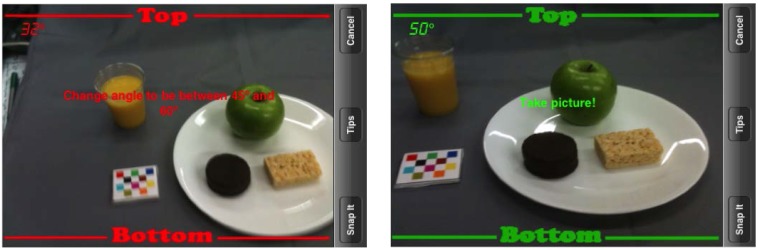
Field of view colored borders that appear when in camera settings of the TADA app to aid with proper angle. A red border (image on left) indicates the camera is not at the correct angle. Children from Samples 1 and 2 were to capture the image when the border was green (image on right). Note the fiducial marker (FM) is the color checkerboard seen of the left of the plate.

The mFR is an application based on one of the technology assisted dietary assessment (TADA) protocols [9,11,12]. Ideally, users are tasked to take an image pair at each eating occasion. Methods of image analysis [13] are used to assess foods and beverages captured in the images. A critical method for image analysis is the inclusion of the FM in the captured image [9,14,15]. The FM is an object of known dimensions and markings, in particular a color checkerboard (noted in Figure 2), that is used for spatial and color calibration of the camera and aids in identification of the foods and beverages and estimation of portion sizes [15].

The TADA app includes parameters to guide users on when to take the image using the embedded information within the mobile device. This is defined by an interchangeable color border (*i.e.*, red or green) shown in Figure 2. Users are tasked to capture the image when the mobile device is held at an angle maintaining a green border by pressing a button labeled, ‘snap-it’. After the image is captured, users are able to view the image and select ‘use’ if the image is acceptable or ‘retake’ (the image) if unacceptable. All captured images are automatically uploaded to a secure TADA website, when a wireless connection is available. Researchers can login to the website to view images among other features. In addition to the system features described here, the devices were preloaded with child appropriate games.

### 2.2. mFR Instructions Provided to Children

Participants were given instruction on how to use the mFR application available on either an Apple iPhone 3Gs running iOS6 (for Sample 1) or an Apple iPod Touch 5^th^ generation (with a rear facing camera) running on iOS7 (for Sample 2). The instruction included a demonstration of launching the application, using the application to capture images (e.g., following the red and green lines in the camera’s view screen, Figure 2), and managing the mFR and FM.

After the introduction instruction, children were given a FM and a colorful silicone wristband to place on the left wrist. The silicone wristband was given to children to guide placement of the FM to the left of the food and/or beverage. Children were asked to wear the wristband for the duration of the time they possessed the mFR. For Sample 2, children were provided four wristbands in the event of misplacing one. Children were asked to demonstrate taking a usable image pair using plastic food replicas, if time permitted or it did not interrupt camp activities. All instructions were provided in an out-of-the-way area within view of camp staff.

During eating occasions at the campsite, researchers and/or camp staff reminded children, ad libitum, to take images of before and after eating, observed the use of the mFR, and assisted children with the mFR as needed. For Sample 1, the mFR, FM, and silicone wristband were collected at the end of the day. The next camp day all were redistributed to a new group of participants until each participant had the opportunity to take, at least, one image of an eating occasion. Children in Sample 2 were assigned two-days to use the mFR before camp started (±2 weeks) and after camp ended (±1 week). When distribution, collection, and redistribution of the mFR and accessories occurred outside of camp, a pre-arranged setting, such as the child’s home or public space (e.g., a mall), was used. Scheduling participants to use the mFR for two-days was dictated by study registration date and availability of the mFR.

### 2.3. Description of the Methods for the Three Defined Skill Sets (Sample 1)

#### 2.3.1. Skill Set 1: Capturing a Usable Image Pair

While in possession of the mFR, all participants were asked to take images of one eating occasion. The camp situation was unique in that the eating occasions were limited to morning snack, lunch, and afternoon snack. Therefore, depending upon the time of the mFR distribution, children had four opportunities to take images during instructions and/or at three eating occasions. To assess the first skill set, a trained human analyst examined before and after images uploaded to the TADA website.

#### 2.3.2. Skill Set 2: Responsibility for the Device

Children were responsible for storing the mFR and FM between and during eating occasions. Camp staff members were trained a priori on how to use the mFR and were informed that children had been asked to independently retrieve the mFR and FM from stored locations during eating occasions.

#### 2.3.3. Skill Set 3: Usability and Acceptability of Using the mFR and FM

Children completed a questionnaire that was interview-administered in an out-of-the-way area within view of camp staff. The questionnaire was comprised of three (3) ‘Yes’ or ‘No’ choice questions. Examples of questions asked were “Was the iPhone (mFR) easy to use for taking pictures of your food and drinks?” and “Would it be easy to carry the checkerboard square (the fiducial marker) around with you?” Copies of the questionnaire can be requested from the corresponding author. If a child answered ‘No’, they were probed to elaborate, e.g., “What did you think was not easy about using the mFR?”, and comments were noted. Similarly, when responses other than ‘Yes’ or ‘No’, were used and the responses better aligned with personal willingness the response was considered ‘Yes’. For example, the phrase, “Maybe if my mom or teacher says it’s ‘okay’”, was considered ‘Yes’.

For participation and cooperation, participants received a $5 gift card upon return of the mFR and completion of the mFR Feedback Questionnaire.

### 2.4. Description of the Methods for the Community Dwelling Sample (Sample 2)

Participants were asked to use the mFR to collect images of all eating occasions over two days at two time points. Each participant was loaned the mFR. Children were instructed to capture image pairs of all eating occasions using an mFR as previously described. Intentionally parents were not given the instructions, unless they asked. The days a child used the mFR varied in that some used it on a weekend day, camp day, or non-camp summer weekday. Observations from Sample 1 informed mFR management methods for Sample 2 and children were given a waterproof carrying case to manage the mFR, FMs, and a charging cord. When the children returned the mFR, researchers asked the children to assist with identifying food items that were indistinguishable, e.g., opaque containers, occluded foods, and to recall foods at eating occasions that were not captured as an image. A trained human analyst examined all images using the TADA server to enumerate and evaluate image pairs. Collectively, these methods were used to assess whole fruit and vegetable intake as part of another study.

For participation and cooperation, participants received $5 and $10 gift cards before and after camp, respectively.

### 2.5. Data Analysis

For this study a trained human analyst examined all images on the TADA server and data were entered into Microsoft Access files specifically designed for each study and then imported into IBM SPSS statistics version 21 for data analysis (IBM Corporation, Armonk, NY, USA). Participant’s responses to the mFR questionnaire provided by Sample 1 were entered and analyzed using the same programs.

For Sample 1, information about each image was entered including date and time stamps, before image or after image, all foods and beverages and/or FM present, and other objects captured in the image. Number of image pairs were examined and assessed as meeting skill set 1 (*i.e.*, FM, all food and beverage, or both present). Statistical examination to compare girls and boys and age group included frequencies, chi-square test, Fisher’s exact test, and independent sample *t*-test. Statistical significance was set at *p* < 0.05 (two-sided).

Data entered for Sample 2 included date and time stamps, image number and order, and amount of whole fruits and vegetables consumed. For whole fruit and vegetables intake, fruit and/or vegetable (100%) juices were excluded [16]. Fruit and vegetable servings/day were calculated by dividing the total fruit and vegetable servings consumed by the total number of days that eating occasions were captured using the mFR. The number of days a child used the mFR was determined by the total number of days the child captured at least one image pair of their food and/or beverage using the mFR. Descriptive statistics were used to describe image pairs. The same statistical tests described under Sample 1 were used for Sample 2. In addition, examination of the number of images within child, between collection periods were assessed using paired *t*-tests.

## 3. Results

A total of 126 children, 3–10 years old, used the mFR. Forty-two were boys and 84 were girls of diverse ethnic backgrounds. The majority of the children were Chamorro (55% Chamorro or 36% Chamorro Mix). The descriptive characteristics of both samples are shown in Table 1. Participants in Sample 1 (*n* = 63) were loaned the mFR over 3–8 h in one day. The second sample of children (*n* = 63) were tasked to use the mFR for two days at two time points.

**Figure 3:**
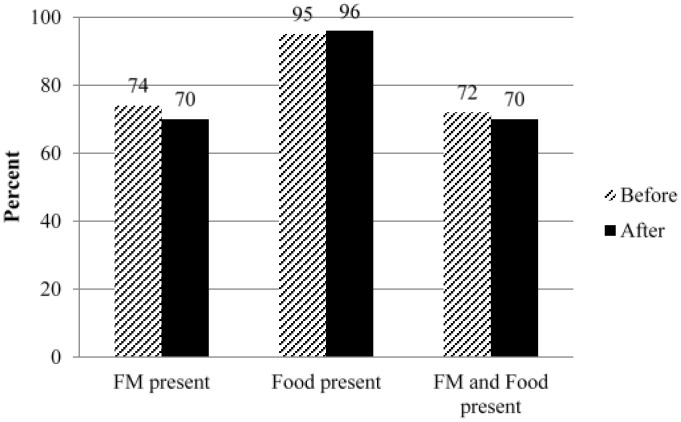
Representation of children’s demonstration of skill set 1 for capturing, at least, one usable image pair (*n* = 57) among Sample 1 in Guam during summer 2013.

Skill set 1 was evaluated in Sample 1. For the task of taking, at least, one image pair of an eating occasion, or the practice image, two image pairs were lost because of technical errors and four image pairs were excluded as four children took only a before or an after image. Therefore, 95% (57/63) were able to demonstrate taking one image pair as shown in Table 2. Of these children, at least 70% (40/57) had the FM present, 95% (54/57) had the food and beverages present, and 70% (40/57) had both in the before and after images (Figure 3). With regard to capturing one image pair, boys were less likely to take an image pair (χ^2^ = 5.755, *p* = 0.026) than girls. Although children were tasked to take only one image pair, some children captured more than one image pair when they possessed the mFR longer (Table 2). Thirty percent (17/57) took two image pairs and 37% (21/57) took three to four image pairs. Of those that took two or more image pairs (*n* = 38), 68% (26/38) were girls and 32% (12/38) were boys. There were no significant differences found between younger (3–6 years) and older (7–10 years) age groups for capturing one image pair and this was also true accounting for more than one image pair. However, boys were more likely to miss including the FM (χ^2^ = 5.216, df = 1, *p* = 0.022), food and/or beverages (χ^2^ = 5.292, df = 1, Fisher’s exact 0.045), or both (χ^2^ = 5.216, df = 1, *p* = 0.022). This significant difference in usable image pairs was primarily due to no after image captured.

**Table 1:** Characteristics of children, 3 to 10 years old, attending summer camp in Guam who used the mobile food record and/or provided feedback in 2013 or 2014.

Characteristics		Sample 1 *n* = 63		Sample 2 *n* = 63	
		*n*	%	*n*	%
Sex					
	Boys	24	38	18	29
	Girls	39	62	45	71
Age groups (years)					
	3–6	27	43	18	29
	7–10	36	57	45	71
Ethnic group					
	Chamorro, only	32	51	37	59
	Chamorro, mixed	24	38	21	33
	Other	7	11	5	8

**Table 2:** Descriptive data of usable image pairs by number of image pairs captured using the mobile food record among children, 3–10 years old, in Sample 1 (*n* = 57 children) attending summer camps in Guam during 2013.

Image Pair(s) ^a^	Total Image Pairs ^a^	FM Present		Food Present		FM and Food Present	
		Before	After	Before	After	Before	After
	57	56	57	50	53	47	51
	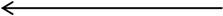 *n* (Percent, %) 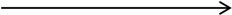						
1	19 (33)	18 (32)	19 (33)	19 (38)	18 (34)	18 (38)	18 (35)
2	17 (30)	17 (30)	17 (30)	15 (30)	16 (30)	15 (32)	16 (32)
3+ ^b^	21 (37)	21 (38)	21 (37)	16 (32)	19 (36)	14 (30)	17 (33)
Total	57 (100)	56 (100)	57 (100)	50 (100)	53 (100)	47 (100)	51 (100)

FM is fiducial marker; ^a^: An image pair is the before and after image for an eating occasion; ^b^: Includes 3 and 4 image pairs.

All children in Sample 1 were able to demonstrate responsibility for the mFR, *i.e.*, skill set 2, as 100% (63/63) returned the mFR undamaged. For the third skill set, all but one child (*n* = 62) completed the questionnaire about usability and feasibility of the mFR and FM. Questionnaire responses by sex are shown in Table 3. Eighty-nine percent (55/62) of participants found the mFR easy to use; 87% (54/62) of children would use the mFR again; and 94% (58/62) reported the FM was easy to carry around. Children volunteered the comment that the FM is “small enough to fit in my pocket”. No differences between boys and girls were found. Among those participants that said the mFR was not easy, when asked why, examples of responses were “it was hard to make it green” (establishing the green border in the mFR camera settings to obtain the correct camera angle), “it was hard to get the board in the image” (fitting the FM in the field of view), and “taking the after eating image”. When asked if they would borrow the mFR again and carry the FM, children who reported, ‘no’, stated “I might lose it”. With regard to carrying the FM a couple of children shared that they would carry it “if it sticks to the iPhone”.

**Table 3:** Responses to mobile food record (mFR) questionnaire among children, 3–10 years old, in Sample 1 after using the mFR (*n* = 62) while at summer camp during 2013.

Statement, As Presented:	Boys (*n* = 23)		Girls (*n* = 39)	
	‘Yes’		‘Yes’	
	*n*	%	*n*	%
The mFR was easy to use	21	91	34	87
I would borrow and use the mFR again	19	83	35	90
Carry the fiducial marker around	21	91	37	95

The community dwelling participants in Sample 2 (*n* = 63) captured 0 to 21 image pairs at Time 1 and Time 2 which were approximately four weeks apart. When the images were reviewed with the children at Time 1 (*n* = 55) and at Time 2 (*n* = 56), five participants reported missing images due to unknown reasons. Technical difficulties aside, 4 of the 63 children (6%) did not take any images at Time 1 compared to 14 of 63 (22%) at Time 2. Two of the children that did not take images at either time point reported only playing the games available on the mFR device. Among those children that took images, the mean number of days were 2.4 (SD ± 1.2) and 2.8 (SD ± 1.1) for Time 1 and Time 2, respectively. The majority of children captured their food and/or beverages using the mFR over, at least, one day at Time 1 (59/63, 94%) and at Time 2 (49/63, 78%). Older participants were more likely to use the mFR longer than younger children as shown in Table 4. However, no statistically significant differences by age and sex were detected with regard to the number of image pairs or the number of days the mFR was used. Seventy-five percent (47/63) of children took images at both time points. Of these children, the mean (±SD) numbers of images captured were 6.21 ± 4.65 for Time 1 and 5.65 ±3.26 for Time 2. There were no significant differences between Time 1 and Time 2 for total number of images and days that the 47 children with repeat measures used the mFR. Estimated servings of whole fruit and vegetables among those children who had images were 0.83 and 0.44 cups per day of fruit and vegetables for Time 1 and Time 2, respectively.

**Table 4:** Descriptive data of images captured using the mobile food record (mFR) among children, 3–10 years old, in Sample 2 (*n* = 63) attending summer camps in Guam during 2014.

Length of Time mFR Used ^a^	Number of Children	Age	Number of Image Pairs ^b^ Per Day	
(Days)	*n* (Percent, %)	Mean (Years)	Mean ± SD	Median (Min-Max)
Time 1				
0	4 (6)	5.5	0	0
1	8 (13)	7.7	1.50 ± 1.07	1.00 (1.00–4.00)
2	20 (32)	8.2	2.28 ± 0.83	2.00 (1.00–4.00)
3	20 (32)	8.2	2.73 ± 1.16	2.42 (1.33–6.00)
4+ ^c^	11 (17)	9.8	2.80 ± 0.87	2.67 (1.33–4.25)
Time 2				
0	14 (22)	7.8	0	0
1	5 (8)	7.8	1.20 ± 0.45	1.00 (1.00–2.00)
2	16 (25)	8.3	2.34 ± 1.29	2.00 (1.00–5.00)
3	18 (29)	8.3	2.07 ± 0.80	1.83 (1.00–3.33)
4+ ^c^	10 (16)	8.0	1.94 ± 0.81	1.75 (1.20–3.75)

SD is standard deviation; ^a^: Number of days at least 1 image pair was captured using the mFR; ^b^: An image pair is the before and after image for an eating occasion; ^c^: Includes image pairs captured over 4 and 5 days.

## 4. Discussion

This is the first study to: (1) evaluate the use of the mFR among young children; and (2) capture self-reported dietary intake among children this age. Results of this study demonstrated that the mFR is potentially a feasible method of dietary assessment in 3 to 10 year olds. Given instructions with demonstration and practice, children 3 to 10 years old were able to use the mFR to record their dietary intakes. Children in both samples were provided unique tasks to capture image pairs, such as at one eating occasion for Sample 1 and any and all eating occasions for a two-day food record over two time periods for Sample 2. The majority of the children exceeded the assigned task in both scenarios. Although responsibility for the mFR was not an objective for Sample 2, it is noteworthy that all participants returned the mFR undamaged as well. Additionally, three-fourths (75%) of children in Sample 2 used the mFR to capture eating occasions at two time points. These results are indicative of their willingness to cooperate and participate. Children in both samples were given the freedom to manage and store the mobile devices where they pleased, such as the carrying cases provided or their lunch boxes. Moreover, every mobile device was returned undamaged which demonstrates that even very young children can be responsible for these devices.

For capturing a two-day food record in a community dwelling situation, children may require reminders and prompts for taking an image pair at all eating occasions. Many children in Sample 2 had used the mFR while at camp where the camp staff were aware of the purpose and knowledgeable on how to use it. When examined by skill set, differences were found only between boys and girls. Children were provided instructions for how to take an image pair for different after eating occasions (e.g., some food leftover or all food eaten). In Sample 1, boys were less likely than girls to take the after eating image, which may suggest the need for an operation incorporated into the mFR to remind children to take after images. One reason this may have occurred is that boys usually ate all of their food. In a previous study [8], adolescents recommended a selection for “ate all food and beverages” in the after image camera setting. This suggestion may address the issue of the missing after images. For the young children included in this study, especially the three to five year olds, attentiveness can be another challenge to remembering to take the after eating image or any image for that matter. Therefore, further research is necessary to determine the age that children can use the mFR independently, as well as the age where a parent or caregiver is needed to prompt and/or assist the child with managing the mFR for taking images.

Age appropriate games were preloaded onto the mFR devices for children to use while they possessed the mFR, which was observed to motivate children to agree to use the mFR and likely maintained engagement for capturing images. Of the children in Sample 2 that did not take any image pairs at either time point, these two children voluntarily disclosed that they used the mFR device for playing games only. These observations corroborate the premise proposed by Lu and colleagues [17] that children’s self-report of diet can be enhanced with animated, customizable agents (e.g., games). The TADA system has the potential to be modified along these lines. On the other hand, some of the age appropriate games were described by the children as being ‘hard to figure out’. Therefore, enhancements to any technology assisted dietary assessment should be designed to be age appropriate in that cognitive abilities, such as literacy level needs to be addressed for children in early childhood (*i.e.*, three to five year olds). For example, the mFR used for studies described in this paper has an automated operation to remind users to include the FM when missing that is a pop-up statement, “fiducial marker is missing [12]”. This enhancement was developed with the input of adolescents [8]. This type of enhancement and any future enhancements for younger children would be better addressed through replacing text with images.

Based on the voluntary comments provided by participants in Sample 1, this study is not without limitations. The challenges that the children reported with regard to using the mFR may be attributed to the small stature of the children and size of their hands. Short-statured children were often observed standing, tip-toeing, or kneeling on a chair to capture the best image. This was also reported by adolescents when they used the mFR [8]. In response to the first reports of issues in achieving the green border, researchers for this study taught children the ‘cup’ and ‘alligator’ holds to improve management and control of the mFR in camera settings given their small hand sizes. These were simple hand positions that symbolized assigned names. With regard to carrying the FM, one child suggested that the FM be fastened to the backside of the mobile device when not in use, but detachable during capturing images at eating occasions. Despite these comments, children still perceived that capturing images with the mFR was easy. In the studies described here, the parents were minimally involved. A large proportion of the community dwelling children were active participants, especially during the first time period. However, a quarter of the children did not complete the second assessment period. Therefore, the right balance between child and parent involvement needs to be examined, as well as methods to generate continuous motivation in the children.

## 5. Conclusions

An image-based dietary assessment method using a mobile device may eliminate the bias of surrogate reporting (e.g., parent or caregiver) of children’s food and beverage intake throughout the day. The children’s high level of technology readiness suggests that dietary methods using technology, like the mFR, may alleviate the burden associated with current dietary assessment methods for children. The significance of this study is that it is the first to evaluate the use of the mFR among young children and moreover, the first study to capture self-reported dietary intake among children this age.
